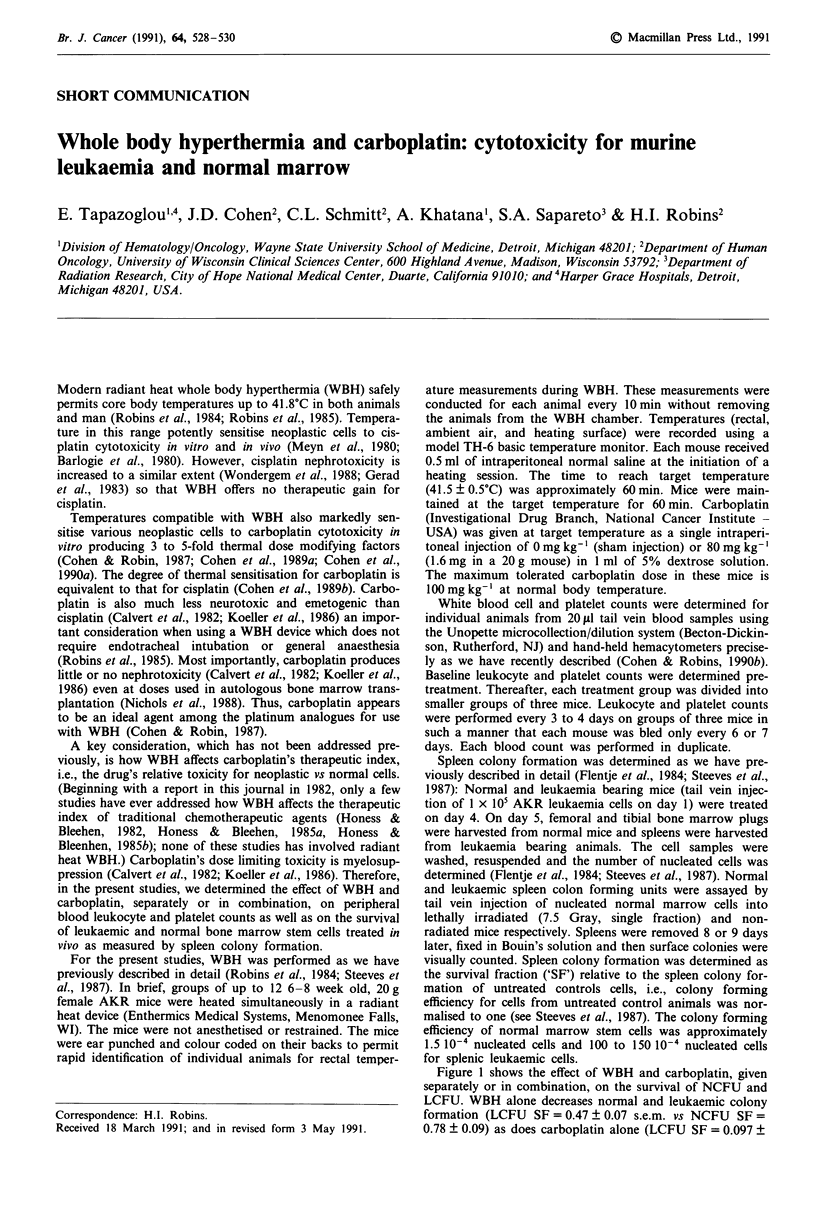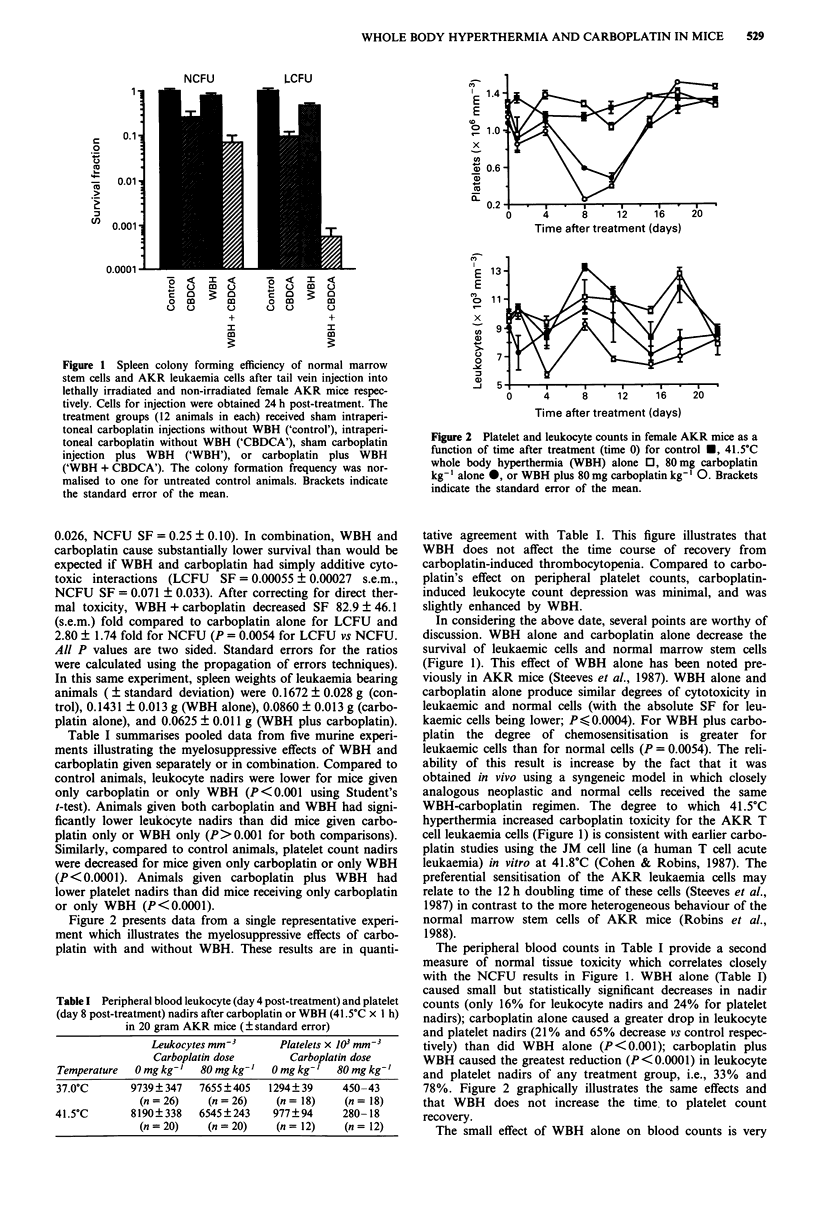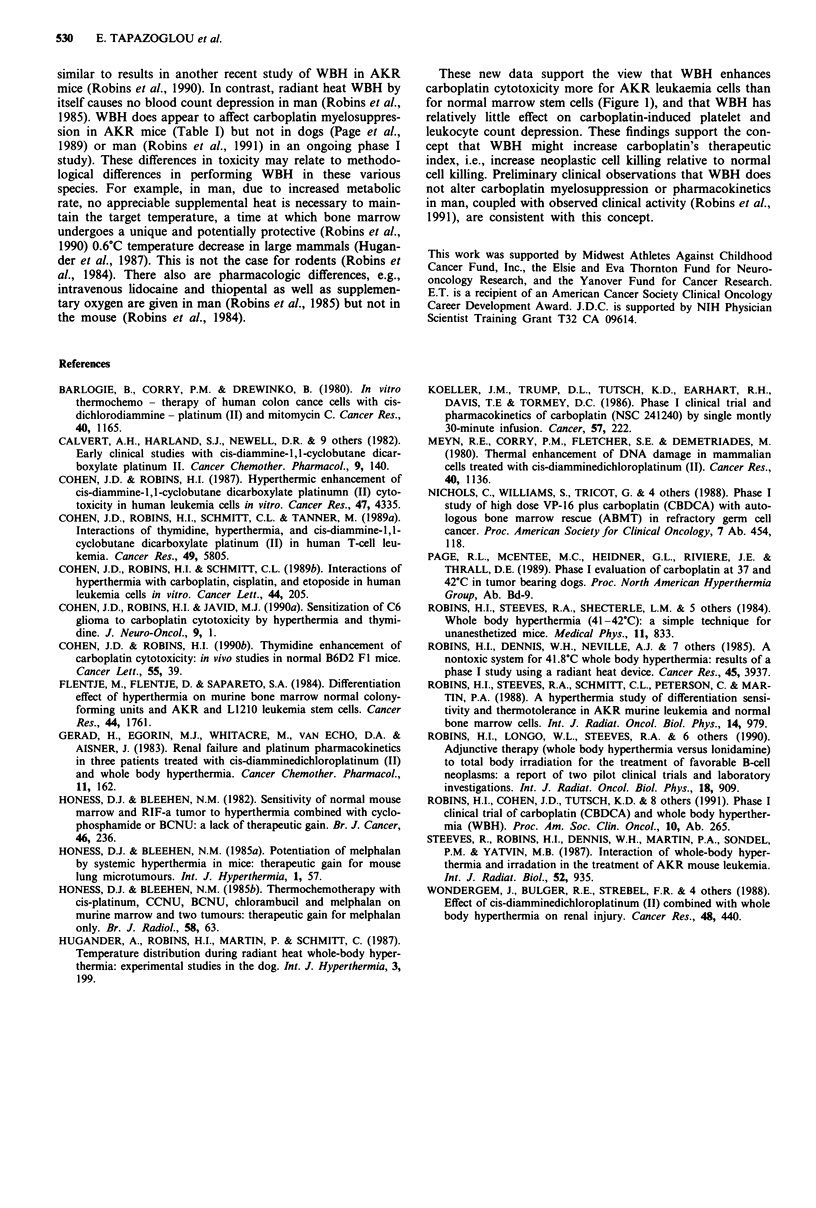# Whole body hyperthermia and carboplatin: cytotoxicity for murine leukaemia and normal marrow.

**DOI:** 10.1038/bjc.1991.343

**Published:** 1991-09

**Authors:** E. Tapazoglou, J. D. Cohen, C. L. Schmitt, A. Khatana, S. A. Sapareto, H. I. Robins

**Affiliations:** Division of Hematology/Oncology, Wayne State University School of Medicine, Detroit, Michigan 48201.


Br. J. Cancer (1991), 64, 528-530 © Macmillan Press Ltd., 1991

SHORT COMMUNICATION

### Whole body hyperthermia and carboplatin: cytotoxicity for murine leukaemia and normal marrow

E. Tapazoglou[1,4], J.D. Cohen[2], C.L. Schmitt[2], A. Khatana[1], S.A. Sapareto[3] & H.I. Robins[2]

[1]*Division of Hematology/Oncology, Wayne State University School of Medicine, Detroit, Michigan 48201;* [2]*Department of Human Oncology, University of Wisconsin Clinical Sciences Center, 600 Highland Avenue, Madison, Wisconsin 53792;* [3]*Department of Radiation Research, City of Hope National Medical Center, Duarte, California 91010; and* [4]*Harper Grace Hospitals, Detroit, Michigan 48201, USA.*

Modern radiant heat whole body hyperthermia (WBH) safely permits core body temperatures up to 41.8°C in both animals and man (Robins *et al*., 1984; Robins *et al*., 1985). Temperature in this range potently sensitise neoplastic cells to cisplatin cytotoxicity *in vitro* and *in vivo* (Meyn *et al*., 1980; Barlogie *et al*., 1980). However, cisplatin nephrotoxicity is increased to a similar extent (Wondergem *et al*., 1988; Gerad *et al*., 1983) so that WBH offers no therapeutic gain for cisplatin.

Temperatures compatible with WBH also markedly sensitise various neoplastic cells to carboplatin cytotoxicity *in vitro* producing 3 to 5-fold thermal dose modifying factors (Cohen & Robin, 1987; Cohen *et al*., 1989*a*; Cohen *et al*., 1990*a*). The degree of thermal sensitisation for carboplatin is equivalent to that for cisplatin (Cohen *et al*., 1989*b*). Carboplatin is also much less neurotoxic and emetogenic than cisplatin (Calvert *et al*., 1982; Koeller *et al*., 1986) an important consideration when using a WBH device which does not require endotracheal intubation or general anaesthesia (Robins *et al*., 1985). Most importantly, carboplatin produces little or no nephrotoxicity (Calvert *et al*., 1982; Koeller *et al*., 1986) even at doses used in autologous bone marrow transplantation (Nichols *et al*., 1988). Thus, carboplatin appears to be an ideal agent among the platinum analogues for use with WBH (Cohen & Robin, 1987).

A key consideration, which has not been addressed previously, is how WBH affects carboplatin's therapeutic index, i.e., the drug's relative toxicity for neoplastic *vs* normal cells. (Beginning with a report in this journal in 1982, only a few studies have ever addressed how WBH affects the therapeutic index of traditional chemotherapeutic agents (Honess & Bleehen, 1982, Honess & Bleehen, 1985*a*, Honess & Bleenhen, 1985*b*); none of these studies has involved radiant heat WBH.) Carboplatin's dose limiting toxicity is myelosuppression (Calvert *et al*., 1982; Koeller *et al*., 1986). Therefore, in the present studies, we determined the effect of WBH and carboplatin, separately or in combination, on peripheral blood leukocyte and platelet counts as well as on the survival of leukaemic and normal bone marrow stem cells treated *in vivo* as measured by spleen colony formation.

For the present studies, WBH was performed as we have previously described in detail (Robins *et al*., 1984; Steeves *et al*., 1987). In brief, groups of up to 12 6-8 week old, 20 g female AKR mice were heated simultaneously in a radiant heat device (Enthermics Medical Systems, Menomonee Falls, WI). The mice were not anesthetised or restrained. The mice were ear punched and colour coded on their backs to permit rapid identification of individual animals for rectal temperature measurements during WBH. These measurements were conducted for each animal every 10 min without removing the animals from the WBH chamber. Temperatures (rectal, ambient air, and heating surface) were recorded using a model TH-6 basic temperature monitor. Each mouse received 0.5 ml of intraperitoneal normal saline at the initiation of a heating session. The time to reach target temperature (41.5 ± 0.5°C) was approximately 60 min. Mice were maintained at the target temperature for 60 min. Carboplatin (Investigational Drug Branch, National Cancer Institute - USA) was given at target temperature as a single intraperitoneal injection of 0 mg $kg^{-1}$ (sham injection) or 80 mg $kg^{-1}$ (1.6 mg in a 20 g mouse) in 1 ml of 5% dextrose solution. The maximum tolerated carboplatin dose in these mice is 100 mg $kg^{-1}$ at normal body temperature.

White blood cell and platelet counts were determined for individual animals from 20 µl tail vein blood samples using the Unopette microcollection/dilution system (Becton-Dickinson, Rutherford, NJ) and hand-held hemacytometers precisely as we have recently described (Cohen & Robins, 1990*b*). Baseline leukocyte and platelet counts were determined pretreatment. Thereafter, each treatment group was divided into smaller groups of three mice. Leukocyte and platelet counts were performed every 3 to 4 days on groups of three mice in such a manner that each mouse was bled only every 6 or 7 days. Each blood count was performed in duplicate.

Spleen colony formation was determined as we have previously described in detail (Flentje *et al*., 1984; Steeves *et al*., 1987): Normal and leukaemia bearing mice (tail vein injection of $1 \times 10^5$ AKR leukaemia cells on day 1) were treated on day 4. On day 5, femoral and tibial bone marrow plugs were harvested from normal mice and spleens were harvested from leukaemia bearing animals. The cell samples were washed, resuspended and the number of nucleated cells was determined (Flentje *et al*., 1984; Steeves *et al*., 1987). Normal and leukaemic spleen colon forming units were assayed by tail vein injection of nucleated normal marrow cells into lethally irradiated (7.5 Gray, single fraction) and non-radiated mice respectively. Spleens were removed 8 or 9 days later, fixed in Bouin's solution and then surface colonies were visually counted. Spleen colony formation was determined as the survival fraction ('SF') relative to the spleen colony formation of untreated controls cells, i.e., colony forming efficiency for cells from untreated control animals was normalised to one (see Steeves *et al*., 1987). The colony forming efficiency of normal marrow stem cells was approximately $1.5\ 10^{-4}$ nucleated cells and 100 to $150\ 10^{-4}$ nucleated cells for splenic leukaemic cells.

Figure 1 shows the effect of WBH and carboplatin, given separately or in combination, on the survival of NCFU and LCFU. WBH alone decreases normal and leukaemic colony formation (LCFU SF = 0.47 ± 0.07 s.e.m. *vs* NCFU SF = 0.78 ± 0.09) as does carboplatin alone (LCFU SF = 0.097 ±

Correspondence: H.I. Robins.
Received 18 March 1991; and in revised form 3 May 1991.

**Figure 1** Spleen colony forming efficiency of normal marrow stem cells and AKR leukaemia cells after tail vein injection into lethally irradiated and non-irradiated female AKR mice respectively. Cells for injection were obtained 24 h post-treatment. The treatment groups (12 animals in each) received sham intraperitoneal carboplatin injections without WBH ('control'), intraperitoneal carboplatin without WBH ('CBDCA'), sham carboplatin injection plus WBH ('WBH'), or carboplatin plus WBH ('WBH + CBDCA'). The colony formation frequency was normalised to one for untreated control animals. Brackets indicate the standard error of the mean.

**Figure 2** Platelet and leukocyte counts in female AKR mice as a function of time after treatment (time 0) for control ■, 41.5°C whole body hyperthermia (WBH) alone □, 80 mg carboplatin $kg^{-1}$ alone ●, or WBH plus 80 mg carboplatin $kg^{-1}$ ○. Brackets indicate the standard error of the mean.

0.026, NCFU SF = 0.25 ± 0.10). In combination, WBH and carboplatin cause substantially lower survival than would be expected if WBH and carboplatin had simply additive cytotoxic interactions (LCFU SF = 0.00055 ± 0.00027 s.e.m., NCFU SF = 0.071 ± 0.033). After correcting for direct thermal toxicity, WBH + carboplatin decreased SF 82.9 ± 46.1 (s.e.m.) fold compared to carboplatin alone for LCFU and 2.80 ± 1.74 fold for NCFU ($P$ = 0.0054 for LCFU *vs* NCFU. *All P* values are two sided. Standard errors for the ratios were calculated using the propagation of errors techniques). In this same experiment, spleen weights of leukaemia bearing animals (± standard deviation) were 0.1672 ± 0.028 g (control), 0.1431 ± 0.013 g (WBH alone), 0.0860 ± 0.013 g (carboplatin alone), and 0.0625 ± 0.011 g (WBH plus carboplatin).

Table I summarises pooled data from five murine experiments illustrating the myelosuppressive effects of WBH and carboplatin given separately or in combination. Compared to control animals, leukocyte nadirs were lower for mice given only carboplatin or only WBH ($P<0.001$ using Student's $t$-test). Animals given both carboplatin and WBH had significantly lower leukocyte nadirs than did mice given carboplatin only or WBH only ($P>0.001$ for both comparisons). Similarly, compared to control animals, platelet count nadirs were decreased for mice given only carboplatin or only WBH ($P<0.0001$). Animals given carboplatin plus WBH had lower platelet nadirs than did mice receiving only carboplatin or only WBH ($P<0.0001$).

Figure 2 presents data from a single representative experiment which illustrates the myelosuppressive effects of carboplatin with and without WBH. These results are in quantitative agreement with Table I. This figure illustrates that WBH does not affect the time course of recovery from carboplatin-induced thrombocytopenia. Compared to carboplatin's effect on peripheral platelet counts, carboplatin-induced leukocyte count depression was minimal, and was slightly enhanced by WBH.

In considering the above date, several points are worthy of discussion. WBH alone and carboplatin alone decrease the survival of leukaemic cells and normal marrow stem cells (Figure 1). This effect of WBH alone has been noted previously in AKR mice (Steeves *et al.*, 1987). WBH alone and carboplatin alone produce similar degrees of cytotoxicity in leukaemic and normal cells (with the absolute SF for leukaemic cells being lower; $P \leqslant 0.0004$). For WBH plus carboplatin the degree of chemosensitisation is greater for leukaemic cells than for normal cells ($P$ = 0.0054). The reliability of this result is increase by the fact that it was obtained *in vivo* using a syngeneic model in which closely analogous neoplastic and normal cells received the same WBH-carboplatin regimen. The degree to which 41.5°C hyperthermia increased carboplatin toxicity for the AKR T cell leukaemia cells (Figure 1) is consistent with earlier carboplatin studies using the JM cell line (a human T cell acute leukaemia) *in vitro* at 41.8°C (Cohen & Robins, 1987). The preferential sensitisation of the AKR leukaemia cells may relate to the 12 h doubling time of these cells (Steeves *et al.*, 1987) in contrast to the more heterogeneous behaviour of the normal marrow stem cells of AKR mice (Robins *et al.*, 1988).

The peripheral blood counts in Table I provide a second measure of normal tissue toxicity which correlates closely with the NCFU results in Figure 1. WBH alone (Table I) caused small but statistically significant decreases in nadir counts (only 16% for leukocyte nadirs and 24% for platelet nadirs); carboplatin alone caused a greater drop in leukocyte and platelet nadirs (21% and 65% decrease *vs* control respectively) than did WBH alone ($P<0.001$); carboplatin plus WBH caused the greatest reduction ($P<0.0001$) in leukocyte and platelet nadirs of any treatment group, i.e., 33% and 78%. Figure 2 graphically illustrates the same effects and that WBH does not increase the time to platelet count recovery.

The small effect of WBH alone on blood counts is very

**Table I** Peripheral blood leukocyte (day 4 post-treatment) and platelet (day 8 post-treatment) nadirs after carboplatin or WBH (41.5°C × 1 h) in 20 gram AKR mice (± standard error)

| | *Leukocytes* $mm^{-3}$ | | *Platelets* × $10^3$ $mm^{-3}$ | |
|---|---|---|---|---|
| | *Carboplatin dose* | | *Carboplatin dose* | |
| *Temperature* | *0 mg* $kg^{-1}$ | *80 mg* $kg^{-1}$ | *0 mg* $kg^{-1}$ | *80 mg* $kg^{-1}$ |
| 37.0°C | 9739 ± 347 ($n$ = 26) | 7655 ± 405 ($n$ = 26) | 1294 ± 39 ($n$ = 18) | 450–43 ($n$ = 18) |
| 41.5°C | 8190 ± 338 ($n$ = 20) | 6545 ± 243 ($n$ = 20) | 977 ± 94 ($n$ = 12) | 280–18 ($n$ = 12) |

similar to results in another recent study of WBH in AKR mice (Robins *et al.*, 1990). In contrast, radiant heat WBH by itself causes no blood count depression in man (Robins *et al.*, 1985). WBH does appear to affect carboplatin myelosuppression in AKR mice (Table I) but not in dogs (Page *et al.*, 1989) or man (Robins *et al.*, 1991) in an ongoing phase I study). These differences in toxicity may relate to methodological differences in performing WBH in these various species. For example, in man, due to increased metabolic rate, no appreciable supplemental heat is necessary to maintain the target temperature, a time at which bone marrow undergoes a unique and potentially protective (Robins *et al.*, 1990) 0.6°C temperature decrease in large mammals (Hugander *et al.*, 1987). This is not the case for rodents (Robins *et al.*, 1984). There also are pharmacologic differences, e.g., intravenous lidocaine and thiopental as well as supplementary oxygen are given in man (Robins *et al.*, 1985) but not in the mouse (Robins *et al.*, 1984).

These new data support the view that WBH enhances carboplatin cytotoxicity more for AKR leukaemia cells than for normal marrow stem cells (Figure 1), and that WBH has relatively little effect on carboplatin-induced platelet and leukocyte count depression. These findings support the concept that WBH might increase carboplatin's therapeutic index, i.e., increase neoplastic cell killing relative to normal cell killing. Preliminary clinical observations that WBH does not alter carboplatin myelosuppression or pharmacokinetics in man, coupled with observed clinical activity (Robins *et al.*, 1991), are consistent with this concept.

This work was supported by Midwest Athletes Against Childhood Cancer Fund, Inc., the Elsie and Eva Thornton Fund for Neuro-oncology Research, and the Yanover Fund for Cancer Research. E.T. is a recipient of an American Cancer Society Clinical Oncology Career Development Award. J.D.C. is supported by NIH Physician Scientist Training Grant T32 CA 09614.

### References

BARLOGIE, B., CORRY, P.M. & DREWINKO, B. (1980). *In vitro* thermochemo – therapy of human colon cance cells with cis-dichlorodiammine – platinum (II) and mitomycin C. *Cancer Res.*, **40**, 1165.

CALVERT, A.H., HARLAND, S.J., NEWELL, D.R. & 9 others (1982). Early clinical studies with cis-diammine-1,1-cyclobutane dicarboxylate platinum II. *Cancer Chemother. Pharmacol.*, **9**, 140.

COHEN, J.D. & ROBINS, H.I. (1987). Hyperthermic enhancement of cis-diammine-1,1-cyclobutane dicarboxylate platinumn (II) cytotoxicity in human leukemia cells *in vitro*. *Cancer Res.*, **47**, 4335.

COHEN, J.D., ROBINS, H.I., SCHMITT, C.L. & TANNER, M. (1989*a*). Interactions of thymidine, hyperthermia, and cis-diammine-1,1-cyclobutane dicarboxylate platinum (II) in human T-cell leukemia. *Cancer Res.*, **49**, 5805.

COHEN, J.D., ROBINS, H.I. & SCHMITT, C.L. (1989*b*). Interactions of hyperthermia with carboplatin, cisplatin, and etoposide in human leukemia cells *in vitro*. *Cancer Lett.*, **44**, 205.

COHEN, J.D., ROBINS, H.I. & JAVID, M.J. (1990*a*). Sensitization of C6 glioma to carboplatin cytotoxicity by hyperthermia and thymidine. *J. Neuro-Oncol.*, **9**, 1.

COHEN, J.D. & ROBINS, H.I. (1990*b*). Thymidine enhancement of carboplatin cytotoxicity: *in vivo* studies in normal B6D2 F1 mice. *Cancer Lett.*, **55**, 39.

FLENTJE, M., FLENTJE, D. & SAPARETO, S.A. (1984). Differentiation effect of hyperthermia on murine bone marrow normal colony-forming units and AKR and L1210 leukemia stem cells. *Cancer Res.*, **44**, 1761.

GERAD, H., EGORIN, M.J., WHITACRE, M., VAN ECHO, D.A. & AISNER, J. (1983). Renal failure and platinum pharmacokinetics in three patients treated with cis-diamminedichloroplatinum (II) and whole body hyperthermia. *Cancer Chemother. Pharmacol.*, **11**, 162.

HONESS, D.J. & BLEEHEN, N.M. (1982). Sensitivity of normal mouse marrow and RIF-a tumor to hyperthermia combined with cyclophosphamide or BCNU: a lack of therapeutic gain. *Br. J. Cancer*, **46**, 236.

HONESS, D.J. & BLEEHEN, N.M. (1985*a*). Potentiation of melphalan by systemic hyperthermia in mice: therapeutic gain for mouse lung microtumours. *Int. J. Hyperthermia*, **1**, 57.

HONESS, D.J. & BLEEHEN, N.M. (1985*b*). Thermochemotherapy with cis-platinum, CCNU, BCNU, chlorambucil and melphalan on murine marrow and two tumours: therapeutic gain for melphalan only. *Br. J. Radiol.*, **58**, 63.

HUGANDER, A., ROBINS, H.I., MARTIN, P. & SCHMITT, C. (1987). Temperature distribution during radiant heat whole-body hyperthermia: experimental studies in the dog. *Int. J. Hyperthermia*, **3**, 199.

KOELLER, J.M., TRUMP, D.L., TUTSCH, K.D., EARHART, R.H., DAVIS, T.E & TORMEY, D.C. (1986). Phase I clinical trial and pharmacokinetics of carboplatin (NSC 241240) by single montly 30-minute infusion. *Cancer*, **57**, 222.

MEYN, R.E., CORRY, P.M., FLETCHER, S.E. & DEMETRIADES, M. (1980). Thermal enhancement of DNA damage in mammalian cells treated with cis-diamminedichloroplatinum (II). *Cancer Res.*, **40**, 1136.

NICHOLS, C., WILLIAMS, S., TRICOT, G. & 4 others (1988). Phase I study of high dose VP-16 plus carboplatin (CBDCA) with autologous bone marrow rescue (ABMT) in refractory germ cell cancer. *Proc. American Society for Clinical Oncology*, **7** Ab. 454, 118.

PAGE, R.L., MCENTEE, M.C., HEIDNER, G.L., RIVIERE, J.E. & THRALL, D.E. (1989). Phase I evaluation of carboplatin at 37 and 42°C in tumor bearing dogs. *Proc. North American Hyperthermia Group*, Ab. Bd-9.

ROBINS, H.I., STEEVES, R.A., SHECTERLE, L.M. & 5 others (1984). Whole body hyperthermia (41–42°C): a simple technique for unanesthetized mice. *Medical Phys.*, **11**, 833.

ROBINS, H.I., DENNIS, W.H., NEVILLE, A.J. & 7 others (1985). A nontoxic system for 41.8°C whole body hyperthermia: results of a phase I study using a radiant heat device. *Cancer Res.*, **45**, 3937.

ROBINS, H.I., STEEVES, R.A., SCHMITT, C.L., PETERSON, C. & MARTIN, P.A. (1988). A hyperthermia study of differentiation sensitivity and thermotolerance in AKR murine leukemia and normal bone marrow cells. *Int. J. Radiat. Oncol. Biol. Phys.*, **14**, 979.

ROBINS, H.I., LONGO, W.L., STEEVES, R.A. & 6 others (1990). Adjunctive therapy (whole body hyperthermia versus lonidamine) to total body irradiation for the treatment of favorable B-cell neoplasms: a report of two pilot clinical trials and laboratory investigations. *Int. J. Radiat. Oncol. Biol. Phys.*, **18**, 909.

ROBINS, H.I., COHEN, J.D., TUTSCH, K.D. & 8 others (1991). Phase I clinical trial of carboplatin (CBDCA) and whole body hyperthermia (WBH). *Proc. Am. Soc. Clin. Oncol.*, **10**, Ab. 265.

STEEVES, R., ROBINS, H.I., DENNIS, W.H., MARTIN, P.A., SONDEL, P.M. & YATVIN, M.B. (1987). Interaction of whole-body hyperthermia and irradation in the treatment of AKR mouse leukemia. *Int. J. Radiat. Biol.*, **52**, 935.

WONDERGEM, J., BULGER, R.E., STREBEL, F.R. & 4 others (1988). Effect of cis-diamminedichloroplatinum (II) combined with whole body hyperthermia on renal injury. *Cancer Res.*, **48**, 440.